# ‘From disaster, miracles are wrought’: a narrative analysis of UK media depictions of remote GP consulting in the COVID-19 pandemic using Burke’s pentad

**DOI:** 10.1136/medhum-2020-012111

**Published:** 2021-03-29

**Authors:** Gilly Mroz, Chrysanthi Papoutsi, Trisha Greenhalgh

**Affiliations:** 1 Zoology, University of Oxford, Oxford, UK; 2 Primary Care Health Sciences, University of Oxford, Oxford, UK

**Keywords:** health policy, narrative medicine

## Abstract

During crises (major events characterised by uncertainty, urgency and threat), society must make sense of rapidly unfolding events. This happens mainly through narrativising—depicting a setting, characters and a meaningful sequence of events and actions unfolding over time. In the early months of the pandemic, UK general practice shifted from face-to-face consultations to a remote-by-default model (telephone, video or e-consultation). This shift was initially widely accepted by press and public, but support waned after a politician declared that the change would be permanent. We invoke Burke’s dramatistic pentad of act, scene, agent, agency and purpose to theorise findings from a detailed analysis of media coverage of the remote-by-default policy and reactions to it. We consider the 12 weeks from March to June 2020 (first lockdown, when remote-by-default services had just been introduced) and 1 week from late July 2020 (following the ministerial announcement). The initial introduction of remote consulting had strong narrative coherence in which all parts of the pentad were balanced: scene (a deadly virus threatening the country) aligned with act (lockdown, including avoiding face-to-face appointments unless essential), agents (the National Health Service and digital technology as heroic macro-actors), agency (general practitioners ‘deployed’) and purpose (to control the pandemic). The later period, however, was characterised by a mismatch between scene (a country emerging from lockdown and resuming normal life), act (imposition of the remote model), agent (a politician known for his enthusiasm for technology), agency (top-down directive) and purpose (modernisation). Whereas media narratives in the first period aligned with the genre of heroic adventure (suggesting a worthy battle, bravely fought), those of the second had characteristics of farce (something both comic and grotesque). We conclude that close reading of media narratives may surface potential misalignments between policy decisions and the context in which they must be implemented.

## Introduction

### Crisis, COVID-19 and narrative

In their book *The Politics of Crisis Management*, Boin *et al* define a crisis as *“a semantic construction people use to characterise situations that they somehow regard as extraordinary, volatile, and potentially far-reaching in their negative implications”* (Boin, Stern, and Sundelius 2016, 145). Crises, these authors suggest, are characterised by three things: uncertainty, urgency and threat. A key element of leadership in times of crisis is sensemaking—producing an account of what is happening, as it is happening, along with a real-time interpretation of its significance (Boin, Stern, and Sundelius 2016).

Coronavirus (COVID-19) was undoubtedly a crisis. By the time WHO declared a pandemic on 11 March 2020, it was already clear that the virus was highly contagious, potentially deadly and spreading rapidly (Adhanom 2020). Governments were exhorted to take urgent steps to contain it, including reducing all unnecessary close contact between humans.

Within 2 weeks, the UK had banned public gatherings; closed pubs, restaurants, schools and churches; required people to stay at home except for essential excursions and asked them to work from home where possible (Prime Minister’s Office 2020). Almost every aspect of the nation’s social and cultural life—including going to the doctor—was profoundly changed. From March 2020, UK general practice introduced a remote-by-default policy, requiring patients to make contact initially by telephone or online form, after which they would be given an appointment for a call-back by telephone or video (NHS England 2020).

Much has already been written about how doctors and other health professionals viewed the shift from face-to-face to remote consultations (Marshall et al. 2020; Peek, Sujan, and Scott 2020; Greenhalgh et al. 2020Gray et al. 2020). In this paper, we consider how the mainstream media reacted to the introduction of a remote-by-default policy. In particular, we explore why a change that was initially framed as necessary and effective was later depicted as damaging and absurd.

Our work draws centrally on narrative theory as applied to policymaking (Fischer 2003; Stone 2013) and media coverage of policymaking (Shanahan, McBeth, and Hathaway 2011). More specifically, we extend theoretical analyses by previous authors who have used Burke’s pentad (explained in the ‘Narrative and the dramatistic pentad’ section) to bring a narrative lens to the study of policymaking (Gusfield 1976; Hajer 2005; Yanow 2015). We return to these previous studies in the ‘Discussion’ section.

### Narrative and the dramatistic pentad

A narrative can be defined as an account of a series of actions and events, unfolding over time, in which characters (of greater or lesser virtue) encounter trouble and strive to resolve or survive it (Bruner 2004). Characters—usually individuals and institutions—act. Acts are meaningful (ie, they have social significance and moral worth), and they are undertaken with motive (the emotionally charged desire to achieve or prevent something).

Narratives may be written—or spoken or enacted—in various formats or genres. A novel, for example, may be presented as fairy tale, science fiction or magical realism; a play may take the form of comedy, tragedy or melodrama. Within these genres, there are literary conventions for what can happen in the story and how (eg, we do not expect a fairy tale to be true but we do expect it to unfold as a fairy tale should). Stone, cited by Shanahan, McBeth, and Hathaway (2011, 374-375), offers various plots for a policy narrative, including ‘story of decline’ and ‘stymied progress’.

Drawing on Aristotle’s writings on the nature of drama, literary theorist Kenneth Burke depicted narrative as concerned above all else with purposeful action towards a goal. He proposed five key elements of any narrative or story: the act (what is done), the scene or setting (the context in which it is done), the agent (who does it), the agency or instrument (how it is done) and the purpose or motive (why it is done) (Burke 1950; Burke 1945). These elements make up Burke’s *dramatistic pentad*—a heuristic tool which helps us work out what is going on in a narrative. When considered in combination, the five elements of the pentad allow us to describe a situation and to understand what is happening and what is causing it to happen— its motive.

The elements in Burke’s dramatistic pentad are closely aligned with what are sometimes known as the ‘five Ws and an H’ in journalism—who (agent), what (act), when and where (scene), why (purpose) and how (agency) (Singer 2008). Burke, however, encourages us to go beyond considering these elements separately and look at the relationships (what he called the *ratios*) between them, thereby uncovering aspects of motive (and, more widely, plot). Burke later added a sixth element (attitude, by which he meant dispositions, beliefs and judgements about the world) to his pentad.

According to Burke, the trouble in a story tends to result from a mismatch between two or more elements of the dramatistic pentad (eg, between the act and the scene). Narrative scholar Jerome Bruner depicted this trouble in terms of disruption of the expected: ‘*…an initial canonical state is breached, redress is attempted which, if it fails, leads to crisis; crisis, if unresolved, leads eventually to a new legitimate order’* (Bruner 2004, 697).

Inspired by Bruner’s reflections on the relationship between narrative and crisis, we decided to apply Burke’s pentad to an aspect of the COVID-19 crisis.

### Remote consultations: trouble in pandemic times

One of the most dramatic changes to health services introduced in the first wave of the pandemic in the UK was the closure of general practices to most in-person visits and the introduction of the system known as ‘total triage’ (NHS England 2020). Patients seeking an appointment with their general practitioner (GP) or practice nurse now had to contact the NHS 111 telephone helpline, telephone the surgery or fill in an online form before being allocated to a call-back or given other advice (eg, go to hospital). This change, which occurred over a period of days towards the end of March 2020 (Gray et al. 2020), was arguably the fastest and most extensive scale-up of a major service innovation ever attempted in the National Health Service (NHS). Whether the patient had symptoms of acute COVID-19 or sought help for a problem unrelated to COVID-19, they were generally assessed by phone, video or e-consultation before (or, more usually, instead of) being invited for an in-person examination (Greenhalgh, Koh, and Car 2020).

As the first wave of the pandemic waned, it was initially assumed that these arrangements would be reversed. Indeed, between March and July 2020 the proportion of GP consultations occurring face-to-face increased from 26% to 50% in England (NHS England 2020). But on 30 July 2020, a government announcement stipulated that GP consultations should remain remote *by default* (Hancock 2020)—an announcement which, as we will see, was not popular with either GPs or patients.

In this study, we sought to examine how the UK mainstream media (national newspapers) covered the remote-by-default policy, and how these narratives changed as the pandemic unfolded. Because we chose to use Burke’s theoretical approach, we have focused primarily on the media texts themselves rather than on the motives of the journalists or the newspapers they were working for.

## Method

The study was part of a wider programme of research on remote consulting during the pandemic, funded by various COVID-19 emergency research funds (listed at end under ‘Funding statement’), and overseen by an external advisory group with patient and lay representation. The media analysis project was developed from a suggestion by lay people on the group who felt it was a crucial element of the national context for remote healthcare.

The online newspaper database LexisNexis was searched for relevant articles in the eight most widely circulated mass-circulation national newspapers. The online version of *The Voice*, described as ‘Britain’s favourite black newspaper’, was also searched to extend the diversity of intended audiences. Search terms used were “GP[s]” combined with each of seven further terms: “video”, “phone”, “telephone”, “remote”, “digital”, “online” and “virtual”. Articles which contained reference to remote GP consultations, whether phone or video, were extracted. The search was repeated for two time periods: period 1 (2 March–31 May 2020), when the incidence of COVID-19 was rising and general practice was introducing and adapting to remote services, and period 2 (30 July–12 August 2020), following the announcement from the Secretary of State that remote-by-default would be long-term policy (although in the event, no articles were identified in the second week in period 2).

We divided our dataset into articles in which the main or a significant narrative was *about* remote consultations and those which made minor reference to remote consultations. We did a preliminary thematic analysis which allowed us to chart and become familiar with the data. While the thematic analysis (reported previously (Mroz et al. 2020)) generated insights about *which* events and topics were covered in the media, it did not allow us to study in depth *how* those events and topics were presented, especially what rhetorical arguments were being made by their authors about causality, motive or the social or moral significance of the changes.

For the analysis reported here, we sought to undertake an explicitly *narrative* study of media articles which would surface the stories being told, including how they were rhetorically and morally framed. We drew explicitly on our contrasting academic and professional backgrounds: XX is a humanities scholar with a PhD in the study of intertextual influences in classical novels and an interest in media narratives; YY is a social scientist with a PhD in technological change; ZZ is an academic GP.

The narrative analysis was undertaken initially through a close reading of all articles by XX, and refined through discussion with other authors, who read the primary dataset in detail. Key to the development of the analysis was discussion among the research team in which we shared and negotiated our different philosophical assumptions and interpretations of the data.

We began by applying Burke’s dramatistic pentad to analyse the narratives in each period by act, scene, agent, agency and purpose—and also consider the relation between these elements. To further the analysis, to Burke’s pentad we added consideration of other narrative elements such as the unfolding of plot and the wider cast of characters. Having identified and analysed each of the five elements, we then looked at each narrative more holistically to better understand the motivation(s). Then, by focusing on one specific element with all its possible ratios, we endeavoured to uncover and analyse the key tensions in each narrative.

The analysis was contextualised with reference to policy documents and key events in the UK’s pandemic response.

## Results

Our search identified 36 articles (19 and 17 in periods 1 and 2, respectively), which included narratives about remote-by-default consulting, and an additional 120 articles (118 and 2, respectively), which referred more tangentially to remote consulting. In the online supplementary appendix 1, we have listed all the articles in the primary dataset and examples of articles from the secondary dataset.

10.1136/medhum-2020-012111.supp1 Supplementary data

The descriptions below relate mostly to the primary dataset of 36 articles but were also strongly reflected in the wider dataset. The 36 articles were drawn from UK broadsheets (*The Times, The Independent, The Telegraph, The Guardian*) and tabloids (*The Mirror, The Mail, The Sun, The Express*) as well as one from *The Voice*. While there were differences in style between the different publications, the narratives in each time period were remarkably similar across all sources, with the main differences being between period 1 and period 2.

Below, we take each time period in turn, describing the elements of the pentad before considering (as Burke recommended) the ratios between the elements and the implications for interpretation of the narrative.

### Period 1 (March–June 2020): the initial narrative of remote-by-default

#### Scene

The scene in this period is clearly one of crisis. From early March 2020, it became evident that COVID-19, while it could be asymptomatic, was a serious and potentially deadly infectious disease which was spreading rapidly in the UK. Over the next few weeks, the number of daily reported cases would surge into the thousands before peaking in May and beginning to fall in June, mirroring a pattern seen in Italy a few weeks earlier (where the death rate was so high mobile mortuaries were seen on the streets). The usual mode of death (respiratory failure, usually after several days spent on a ventilator) was particularly unpleasant and frightening. In high-incidence areas, hospitals were quickly overwhelmed with breathless patients; one was reputed to have run out of oxygen (Marsh and Booth 2020). General practices were inundated with people phoning up with symptoms of suspected COVID-19, concerned that they would deteriorate and die. While these patients had significant health needs, they were also highly contagious and a danger to both staff and fellow patients. To reduce infection risk, a new system of ‘hot hubs’ was established to handle potentially infected patients, including in some cases examining them in roped-off corridors, outdoor car parks or temporary structures such as tents. Personal protective equipment, including masks, gowns, gloves, protective goggles and overshoes, became the NHS uniform—but was in short supply. Despite precautions, the number of cases rose inexorably. Thousands of frontline healthcare workers became sick from the virus and hundreds died. The public was encouraged to stand on their doorsteps once a week and ‘clap for our carers’ in a grim acknowledgement of the challenges and risks faced by NHS staff.

The scene for the introduction of remote consulting was thus fast-moving, chaotic and dangerous. A military theme depicting a country (and, more specifically, NHS) under heavy and sustained attack from a dangerous enemy (the new coronavirus SARS-CoV-2) runs through many of the articles in our period 1 dataset. One article, written by a former military doctor now serving as a GP, is entitled *“I’m running my surgery like it’s the frontline in a war zone”*:

The 53-year-old said: The country is on a war footing and we are seeing people coming together in a fantastic way in the spirit of the Blitz.

It has never gone away from the British psyche.

Look at Thursday night as a great example of it.

People are applauding the troops, and right now the troops are NHS workers on the frontline.

(*The Sun*, 3 April, article from wider dataset)

#### Act

Articles in this period covered two closely linked acts. The first was the rapid move to total triage and remote consulting in general practice, driven by NHS England, which began in early March 2020 (NHS England 2020). Patients had to phone their GP surgery or book an appointment online and have their consultation remotely, usually via telephone and sometimes via video. This change was depicted by newspapers as timely and necessary. The second (linked) act was the far-reaching imposition of lockdown by the government on 23 March 2020 in an effort to stop the spread of the virus. In an announcement which came as a surprise to some but which was later criticised as occurring too late, people were ordered to stay in their homes except for essential trips, and a third of the workforce was furloughed (Prime Minister’s Office 2020). Remote healthcare was thus part of a wider set of unprecedented but necessary measures which were proportionate to the deadly and advancing threat. For the first time since the establishment of the NHS in 1948, it was no longer possible to walk into a GP surgery and ask to be seen—but this was also the first time in living memory when schools were closed to most pupils, people were being paid not to work and weddings were banned.

#### Purpose

The primary purpose of the shift to remote consulting, as with the wider aspects of lockdown, was of course to ‘*reduce the spread of COVID-19’* (*The Independent*, 16 March) by ‘*reduc[ing] the risk of infected patients turning up at surgeries’* (*The Daily Mail*, 10 March). Consulting remotely would keep doctors and patients safe, and would, according to articles published at the time, ‘*free GPs to deal with the extra workload created by the virus’* (*The Guardian*, 6 March).

#### Agent

Articles published in this period depict a number of characters—both macro-actors and individuals—whose roles align with the dominant military theme. Macro-actors in the war include COVID-19 (repeatedly portrayed as a powerful enemy invader who is advancing and doing great damage), the NHS (and especially hospitals and their staff, portrayed as fighting back heroically), and—in several articles—digital technologies, which were depicted not merely as tools but, along with the technology companies, as agents in their own right, as illustrated by the headline ‘*Digital front opens in war on disease as start-ups come to the aid of NHS’* (*The Times*, 29 March).

Individual actors in this narrative include GPs, portrayed as frontline soldiers whose contribution will help ‘*fight the spread of coronavirus’* (*The Daily Mail*, 10 March) and as receiving commands from higher up the ‘military’ hierarchy, as in ‘*GPs told to switch to digital consultations to combat COVID-19’* (*The Guardian*, 6 March). The depiction of GPs in passive and subordinate terms aligns with a previous analysis we undertook comparing media depictions of hospital doctors and GPs: whereas the former were depicted as heroic, knowledgeable and virtuous, the latter tended to be portrayed as lacking in skills, work-shy and motivated by money (Barry and Greenhalgh 2019).

In the period 1 narrative, patients are depicted (for the most part) as background characters—passive civilians in the ‘war’. One or two articles in our dataset were directed at patients, conveying commands to them about what to do in the crisis. The *Telegraph*, for example, advised people to ‘*Ring GPs, don’t visit’* (15 March); the style resonates somewhat with government advice given to civilians during the World War II, which emphasised staying at home and contributing in small and prescribed ways to the war effort (eg, ‘dig for victory’, ‘make do and mend’).

#### Agency

While it might be expected that technology would be depicted as the tool through which GPs (agents) would fight the battle against COVID-19, and this was the case in some articles, we also found that the principal agents were often depicted as technologies and technology firms while GPs were depicted as tools. The following extract, for example, depicts GPs as part of a workforce being ‘deployed’ by digital macro-actors:

Earlier this month, the NHS ordered England’s 7000 GP surgeries to conduct as many patient consultations as possible by video connection to help reduce the spread of COVID-19. […]. As more people self-isolate, we are helping COVID-19 positive patients maintain ready access to GP care at home and minimising the spread of infection, says [managing director of technology company]. […]. [D]igital appointments help to deploy the workforce more efficiently by making it easy for GPs to work flexibly and remotely.

(*The Independent*, 16 March)

#### Analysis of motive

By considering what Burke called the ratios between different elements in the pentad, the motivation of the situation can be discerned. The clear motivator of the narrative is the scene: indeed, without the pandemic, none of the other elements would be required (there would be no story). As Burke noted, ‘*[i]t is a principle of drama that the nature of acts and agents should be consistent with the nature of the scene’* (Burke 1945, 3).

With the dramatic crisis scene set, it is also evident that all elements are interconnected in their causality:

- Scene-purpose: without the scene (the rising pandemic along with lockdown), there would be no purpose (to slow the spread of the virus).
- Purpose-act: without the purpose, there would be no act (moving to remote care).
- Act-agent: without the act, digital technology and technology companies would not be the agent of care provision.
- Agent-agency: without technology as agent, GPs would not have become the means by which remote care would come to be implemented.

**Figure 1:**
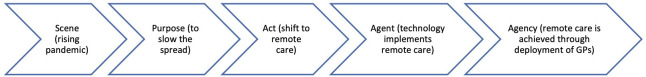
Narrative causality in period 1. GP, general practitioner.

In this way, the first element in each binary can be considered the direct cause of the second, with the scene setting the stage for the presence of each element in the development of the narrative (figure 1).

#### Tensions with technology as agent

While the narrative in period 1 has a great deal of coherence, there are nevertheless some tensions which store up trouble for the future. The most controversial element in this pentad is the role of technology as agent. This is partly due to a key feature that is unusual for the protagonist of a narrative—its inanimateness. But it is also due to the tensions that arise in its relationship with the other elements in the pentad.

First, there were agent-act tensions. The act of shifting to remote care enabled digital technology to become the agent of the narrative, but this was not without controversy. Even at a time when digital technology was being depicted as a potential revolutionary hero, the clash of interests between private-sector technology firms and the public-sector NHS was evident. The pandemic was not merely a war which technology could help win but a *commercial opportunity* (veiled in a narrative of reassurance directed towards patients) for digital start-ups (*The Independent*, 15 April).

There were also agent-purpose tensions. The purpose projected by the narrative was to reduce the spread of coronavirus (implicitly, to keep people safe and prevent deaths). One of the striking features of the early articles was their focus on new digital technologies as the agents through which the purpose would be achieved. There was little mention, for example, of the old-fashioned telephone—yet in reality most remote consultations in England from March to June 2020 occurred by phone (NHS England 2020). Thus, as narrativised in the mainstream media, the purpose (controlling the pandemic) motivates not the *act* (the shift to remote consultations) so much as the *agent* (new digital technology).

The most awkward tension surrounding the role of technology as agent is perhaps the agent-agency tension: reduction of GPs to the *means by which* digital healthcare acts. As the quote above about digital technology ‘deploying’ GPs shows, rather than depicting GPs as frontline heroes of the war on the virus, the narrative at times paints them as subordinate to technology. One commercially provided video consultation service is described as one which ‘*allows GPs and other healthcare professionals to care for people at home via digital consultations’* (*The Independent*, 15 April, our emphasis; see also quote from *The Independent* 16 March above). The wording of this quote evokes the image of a master and subordinate: without the support, direction and permission of the former (the digital technology provider), the latter (the healthcare professional) is incapable of acting.

In this early narrative, there is also a hint of agent-scene tension. GP support for remote consulting was due largely to the backdrop of the pandemic, which rendered the move ‘necessary’ (*The Guardian*, 19 April). But they also looked to the future, to a post-COVID-19 scene: one GP suggested that ‘*[d]igital healthcare, if done well, has a way of creating positive change’* (*The Voice*, 9 April), while another spoke of how ‘*[e]ven after the pandemic, up to half of GP appointments would be online or by telephone’* (*The Times*, 4 April). These statements begin to set a new scene for the act, and seem to offer positive though cautious support. But while GPs describe the move to remote consulting it as ‘sensible’ in the context of the threat of COVID-19 (*The Guardian*, 6 March), they also comment that remote consultations are *“not suitable for all patients”* (*The Daily Mail*, 2 May). These guarded and qualified comments betray an important attitude (Burke’s sixth element of narrative) among GPs that the act-scene ratio is likely to change before long, and that they do not view the future simply as a continuation of the present.

#### The seeds of trouble

In the early weeks of period 1, the narrative builds a dramatic story: in a scene of escalating crisis, digital technologies for remote consultations emerge as the hero that will help fulfil the purpose of reducing the spread of COVID-19, thereby saving lives—a narrative which aligns well with the wider narrative prevailing at the time of the heroic NHS (especially hospitals) bravely fighting the pandemic. From the middle of period 1 (around late April) onwards, a slight shift occurs in the scene: the pandemic is still present, but daily case numbers are no longer rising and appear to be coming under control. Lockdown remains in place; people are still required to stay at home and consult their GP remotely (act) in order to reduce the spread of the virus (purpose). But this state of affairs has, at least temporarily, become canonical and hence is no longer a reportable story. The main characters (digital technologies) and the means by which they fight the war (GPs) both fade from the narrative. In their place emerges a new group of characters—patients—who have heretofore been bystanders in the drama. Early articles referred to patients in generic ways but kept them backgrounded and depicted them as receiving commands, but gave them little voice of their own. This changes at the end of May, when several articles feature patients more prominently describing their experience of remote consultations.

Patients’ accounts of remote care in this period are mostly positive. One woman, for example, describes her experience of a video consultation for a pimple in her armpit as *“the most painless doctor’s appointment of my life […]”* (*The Telegraph*, 22 May). This patient adds a dramatic coda—‘*From disaster, miracles are wrought’*—to illustrate how, in her view, the pandemic has provided the impetus for positive technological change. Thus, satisfied patients reinforce the technology-as-superhero narrative.

However, one negative experience is also recounted. The patient describes how he:

[…] deteriorated rapidly […] after speaking to two different GPs on the phone […] Both GPs missed my coronavirus symptoms despite me saying I had an excruciating headache, hot and cold sweats, loss of appetite and was constantly coughing. (*The Telegraph*, 25 May)

The emergence of patient narratives about possible serious risks of remote consultations is a major twist in the plot. While GPs had expressed caution throughout about the *hypothetical* risks of consulting remotely, these initially seemed footnotes in the story. The quote above reveals two aspects of trouble: first, that state-of-the-art digital technology is not being widely used (the consultation was by telephone) and second, an important diagnosis, which eventually required hospitalisation, has allegedly been missed. Technology may initially have been heralded as the saviour during the pandemic, but the patient narratives begin to reveal that it has not quite lived up to expectations. The hero’s fall from grace thus begins. As we shall see, it is soon accelerated by an event nobody had predicted. Instead of an epilogue, we will need a sequel.

### Period 2: remote-by-default as the unwanted ‘new normal’

#### Scene

By July 2020, and following a substantial number of deaths and a major blow to the economy, the pandemic had receded in the UK, and as a result lockdown measures were gradually lifted. The military theme is no longer present in media narratives, which suggests that the war against coronavirus is no longer considered to be waging (and, perhaps, that it is assumed to have been won). General practice, while far from business as usual, had achieved what felt to be a significant milestone, with half of all consultations occurring face-to-face once again (NHS England 2020).

#### Act

Despite the easing of lockdown and the gradual return to face-to-face GP appointments throughout July, on 30 July the Secretary of State for Health, Matt Hancock, announced in a speech at the Royal College of Physicians that ‘*from now on, all consultations should be teleconsultations unless there’s a compelling clinical reason not to’* (Hancock 2020). In other words, people were required to continue to stay at home and consult their GP remotely. Interestingly, while the now-familiar military metaphor was not used directly by the press, it is present in Mr Hancock’s speech as quoted in the articles (eg, *“the pandemic has been as close as you can get to fighting a war without actually fighting a war”*). Thus, the scene selected by the Secretary of State to justify the act is not the current one but one which has just passed.

#### Purpose

As covered by the media articles in our period 2 dataset, the main purpose for the remote-first policy was not to keep people safe, but rather to modernise an outdated NHS in which clinicians were set in their inefficient traditional ways. *The Telegraph*, for example, reported that ‘*there had been dramatic changes to how the NHS worked as a result of the pandemic and it could not be allowed to “fall back into bad old habits”’* (*The Telegraph* (a), 31 July). The aim of the new policy, reported another article using a direct quote from the Secretary of State’s speech, was to *“take what we have learnt and build back better”* (*The Times* (a), 31 July).

An NHS that still uses 9000 fax machines and 10 per cent of the world’s remaining pagers is manifestly overdue for the sort of digital reckoning set out by Matt Hancock, the health secretary, in a speech yesterday. Now that the first peak of the coronavirus pandemic and the acute pressure it brought to bear on the NHS has passed, ministers and clinicians have an opportunity to apply lessons learnt and look to the future. If Mr Hancock has his way, they will do so via webcam.

(*The Times* (b), 31 July)

#### Agent

A number of changes have occurred in the character list from the previous time period. Perhaps the most significant is the entrance centre stage of a new character, Matt Hancock, who remained mostly in the background in period 1. As Secretary of State, his role is one of a senior policymaker pursuing an explicitly political agenda of a digital revolution in the NHS. In all the articles identified for this short period 2, he is the protagonist, and agent, of the act—and indeed, usually depicted as a villain in the story because of the threat his new policy was seen as posing to safe patient care.

This means that despite the story being *about* technology, technology is no longer the agent. Indeed, technology is not even a main character. It is now a fallen hero, whose demise began in the middle of period 1 when it starts to fade from the narrative. Technology’s continued fall from grace in part 2 coincides with the retreat into the background of two other original key characters: COVID-19 (the conquered war villain) and the technology companies who had developed novel digital solutions (who have no special role if bespoke remote solutions are no longer needed).

While these characters remain largely off stage, a group of characters that first made their presence felt at the end of period 1—patients—make another appearance. Whereas in period 1 patients were usually satisfied customers and (on only one occasion) wronged victims, the patients in period 2 are *mostly* victims who come to harm (or narrowly escape it).

Another group whose characters undergo a change is GPs. In period 1, they were frontline soldiers battling coronavirus or the instruments to be ‘deployed’ by technological macro-actors. In Mr Hancock’s speech, they are foot-soldiers responsible for implementing his policy. But in the media articles, they are afforded agency of their own as concerned professionals raising doubts (described in the ‘Agency’ section) about patient safety.

#### Agency

The instrument through which Mr Hancock wishes to implement a remote-first service is, of course, his policy directive. However, the role of agency (and, relatedly, of agents) becomes more complicated, and ambiguous, when GPs and technology are added.

- Mr Hancock uses the instrument (policy directive) to implement the act (sustaining remote-by-default GP consultations).
- The policy directive, in turn, uses GPs to implement the act.
- GPs use technology to implement the act.

Mr Hancock remains the only sole agent. His policy directive and GPs are both agents and instruments. Technology, in contrast to its role in period 1, is now only a means: rather than implementing the act, it is now the instrument through which the act is being implemented.

According to Burke, the ambiguity regarding the function of certain characters, such as GPs in this case (are they agent or agency?), should be welcomed. Indeed, his intention of the pentad is not to create ‘*terms that avoid ambiguity, but terms that clearly reveal the strategic spots at which ambiguities necessarily arise’* (Burke 1945, xviii).

It is also worth noting the way technology elides from agent (a character in the story who does things and has virtues) in period 1 to agency (a tool or mechanism) in period 2. Rather than specific, newly developed bespoke digital solutions with implicit agency of their own, Mr Hancock is depicted as promoting the use of generic technology as the new *modus operandi* of general practice—for example, video consultations as ‘*Zoom medicine’* (eg, *The Independent*, 1 August), or encouraging doctors to use the generic application WhatsApp ‘*to speak with both colleagues and patients’* (*The Independent*, 30 July). Technology is no longer the revolutionary hero stepping forward to lead the pandemic response; it has been recast in the role of instrument.

#### Analysis of motive

**Figure 2:**
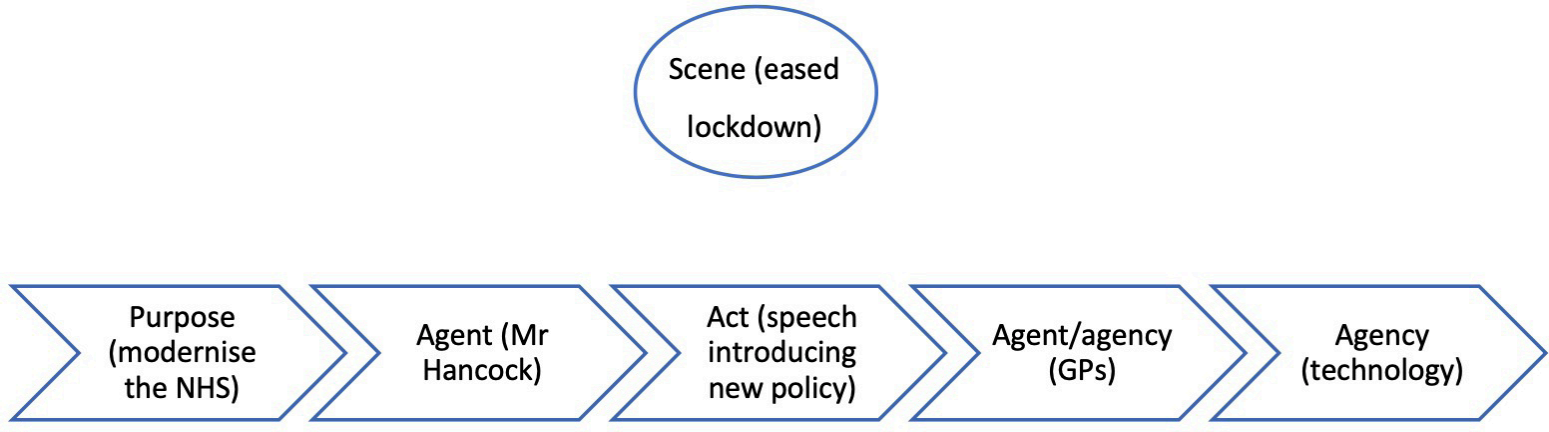
Summary of the narrative causality in period 2. GP, general practitioner; NHS, National Health Service.

In part 1, the scene (the rising pandemic) was the clear motivation behind the purpose (to reduce the spread of coronavirus), which in turn clearly motivated the act (the shift to remote consultations). But in part 2, the scene (eased restrictions) is unrelated to both the purpose (modernising the NHS) and the act (implementing a remote-first policy). This narrative is depicted in figure 2.

Mr Hancock’s announcement is at odds with the scene: why did he decide to implement his policy at a time when the pandemic had receded and GP care was returning to normal? The answer is that his act was motivated by his purpose (modernising the NHS), which serves as the dominant driving force behind the narrative.

The motivations in the narrative can be summarised as follows:

- The purpose (modernising the NHS) causes the agent (Mr Hancock) to act (declaring the policy directive in his speech).
- The act (the speech) causes GPs (agent/agency) to use technology (agency).

It is worth reiterating here Burke’s observation that ‘*[i]t is a principle of drama that the nature of acts and agents should be consistent with the nature of the scene’* (1945, 3). The absence of the scene from the narrative chain in this sample of articles is therefore striking, as it is unusual in narrative for this element to have so little impact on the act and agent, and therefore the narrative.

However, there are two genres of narrative which, according to Burke (1945, 3), ‘*may deliberately set these elements at odds with one another’*—the comic and the grotesque. These can be combined in the genre of farce, which dictionary.com defines as ‘*a comic dramatic work using buffoonery and horseplay and typically including crude characterisation and ludicrously improbable situations’*. It is the stark misalignment between the scene (the steady return to face-to-face care) and the act (imposing remote-by-default care), which tilts the narrative to the genre of farce and even satire—that is, the use of humour, irony or ridicule to expose and criticise the vices or folly of others, especially in the context of contemporary politics. We illustrate this shift in genre in the next section.

#### Tensions: the scene

The mismatch between the scene and the remaining elements is the narrative’s most evident and awkward tension. Whereas in part 1 the scene served as the primary motivator of the narrative, in part 2 it is completely unrelated, thus painting the denouement in a questionable light.

Media articles in period 2 make much of the scene-act tension. The act (a speech-act declaration that all GP consultations should be remote by default) is dramatically at odds with the scene (easing of lockdown). Indeed, while ‘*[a]t the height of the COVID-19 epidemic it was understandable that GPs should try to avoid face-to-face contact where possible’* as it was ‘*vital the disease was contained’* (*The Express*, 31 July), the scene has now changed (the virus is contained), and the act—according to these articles—is no longer needed. The mismatch between the scene and the act is strengthened by the fact that even ‘*throughout the pandemic face-to-face appointments have been facilitated when they’ve been necessary’* (*The Guardian*, 30 July).

The pandemic has receded, with many services (including GP care) having returned towards normal, and negative experiences among patients (and also among doctors) continue to accumulate. These call the speech act into question and cast doubt on its underpinning assumption (a remote-by-default service is fit for the present and future as well as the past). The articles in period 2 list various limitations of remote GP consultations, including the possibility of missing serious illness, potential threats to effective communication and the GP-patient relationship, and questions of accessibility to those lacking digital connectivity and devices. One article quotes a GP as summing up the situation thus: ‘*[r]emote consultations is undeniably a riskier way to practice medicine and, while accepted during the COVID-19 pandemic, it is vital that there are checks and balances in place’* (*The Independent*, 30 July).

Patient narratives in period 2 continue to include both positive and negative experiences, but the latter (which now extend to video as well as phone consultations) outweigh the former in both frequency and rhetorical power, especially the ‘lucky escape’ plot. In an article titled ‘*GP video calls aren’t all they’re cracked up to be’*, a patient describes her video consultation for a facial lesion, which the GP diagnoses as a (harmless and self-limiting) cold sore. Unconvinced, the patient goes to see (in-person) a private clinician, who diagnoses the lesion as a precancerous growth (*The Independent*, 4 August).

In period 1, remote-by-default consultations were depicted in articles not as a generic, for-all-time solution but as a necessary trade-off between the canonical (life as usual) and the constraints of the crisis (the threat of the virus), although it was recognised that some remote consultations could be acceptable and useful in the future. As depicted in the articles in our period 2 dataset, there is now no logical explanation for the policy change—the scene does not require it. Instead, through his act, Mr Hancock appears to be endeavouring to resurrect the fallen hero—technology—oblivious to the negative reactions from those whom his act directly affects. He appears to dismiss the widespread concern among GPs that remote consultations are ‘*certainly not the wonder drug to cure the ills of General Practice’* (*The Independent*, 31 July).

This unresolvable mismatch between act and scene brings the narrative into the genre of farce, to which patient stories add a distinct satirical edge. The aforementioned patient with the (allegedly) missed precancerous lesion, having been reassured by the private physician that her condition was ‘*nothing to panic about immediately’*, concludes her narrative by drawing attention to a concern that received limited attention when the remote-first policy was drawn up:

In any case, what happens if one needs to discuss matters gynae. Have you ever tried taking a photo of your nethers? Not everyone is a contortionist. Or are we all going to be issued with long-handled NHS selfie sticks?

By adding comedy to her account, the patient draws attention to the farcical nature of the situation. Hidden behind the humour is a serious concern regarding both the remote-first policy and Mr Hancock’s insistence on its implementation: there are many kinds of health consultation (regardless of the scene) which simply cannot, and should not, be done remotely.

There are also scene-purpose tensions. One journalist declared that Mr Hancock is on a ‘*crusade to introduce more digital technology to the NHS’* (*The Express*, 31 July). In this article, the word ‘crusade’ is synonymous with the agent’s purpose to modernise the NHS through digitisation.

According to dictionary.com, ‘crusade’ has three definitions: (1) ‘*any of the military expeditions undertaken by the Christians of Europe in the 11th, 12th, and 13th centuries for the recovery of the Holy Land from the Muslims’*; (2) ‘*any war carried on under papal sanction’* and (3) ‘*any vigorous, aggressive movement for the defence or advancement of an idea, cause, etc’.*

These definitions suggest, perhaps, that (1) Mr Hancock’s policy is incompatible with the current times; (2) the policy was introduced based on the Secretary of State’s personal beliefs, and had hints of an assumption of Divine right and (3) it was implemented vigorously and aggressively, without discussion with those most affected by it as to how they would like to proceed.

By framing Mr Hancock’s purpose as a ‘crusade’, the journalist thus highlights its incompatibility at a time when society is longing to continue its return to normality.

Finally, there are scene-agent and scene-agency tensions. The sudden appearance of the agent, Mr Hancock (the country’s most senior policymaker in health), in part 2 at a time when the scene (the receding pandemic) does not urgently require it, contrasts with his relative obscurity in part 1, at a time when the scene (the rising pandemic) would have made his appearance appropriate. The Secretary of State is on stage when he should not be, whereas previously he was not on stage when he could have been. He is thus out of place, and furthermore, the *agency* (GPs using technology to implement the policy) is also out of place.

The counter-narrative, voiced by both GPs and patients, of remote consultations as risky, inequitable and sometimes inappropriate (and even so impossible as to be comical) further establishes digital technology as a fallen hero that has not lived up to expectations. Mr Hancock rejects this recharacterisation of technology, and while he now appears to place technology in the role of agency (instrument) rather than agent (actor), he nevertheless comes across as clinging to a belief that technology, in some form or another, can be implemented to transform the NHS into the digital utopia he desires it to be.

## Discussion

In this narrative analysis, we considered media depictions of policy interventions around the shift from face-to-face GP consultations to telephone or video. We used Burke’s dramatistic pentad as our main theoretical lens to compare two periods: March–June 2020, when remote-by-default services had just been introduced as part of wider lockdown measures, and a week from late July 2020, following a ministerial announcement that remote-by-default GP consultations would remain after the pandemic ended. Analysis revealed that media stories of remote consulting in period 1 (the original drama) had strong narrative coherence in which all elements of the pentad were balanced and made sense: scene (a deadly virus threatening the country) aligned with act (lockdown, including avoiding face-to-face encounters unless essential), agents (the NHS and digital technology as heroic macro-actors), agency (GPs ‘deployed’) and purpose (to control the pandemic), although there were some tensions—particularly around the depiction of technology as agent. In period 2 (the sequel), however, articles depicted a mismatch between scene (a country emerging from lockdown and resuming normal life), act (imposition of the remote model), agent (a politician known for his enthusiasm for technology), agency (top-down directive) and purpose (modernisation of the NHS). In this second period, there were multiple tensions, most notably between scene and act.

Whereas media narratives in the first period aligned with the genre of heroic adventure (suggesting a worthy battle, bravely fought), those of the second had characteristics of farce (something both comic and grotesque)—a fitting genre to present political satire.

Our study has demonstrated a more general methodological finding—that close reading of media narratives, undertaken as an interdisciplinary study between scholars of the humanities, the health sciences and the social sciences, may surface misalignments between health policy decisions taken during a crisis and the turbulent context in which they must be accepted and implemented.

Some scholars have depicted policymaking as fundamentally a narrative process involving storytelling, rhetoric and enacted drama (Fischer and Forester 1993; Majone 1989; Stone 2013; Turner 1980). These authors argue that policymaking is not, as is generally assumed, an essentially technocratic process of obtaining evidence to solve a pre-existing problem, making decisions and implementing them. Rather, it is fundamentally an interpretive and discursive process: *framing* a problem, *negotiating* its meaning and *arguing* for one or other solution to it.

policy-making is a constant discursive struggle over the criteria of social classification, the boundaries of problem categories, the intersubjective interpretation of common experiences, the conceptual framing of problems, and the definitions of ideas that guide the ways people create the shared meanings which motivate them to act (Fischer and Forester 1993, 2).

Despite the undoubted narrative turn—and a strong focus on argumentation—in some areas of policy analysis over the past few decades, few such analyses have drawn explicitly on literary theory. In a literature search, we found only a handful of previous studies which used Burke’s pentad to analyse the policy-making process or its reception by society. Gusfield (1976) used the pentad to inform a sociological analysis of how scientific research comes to inform (or not) drink-driving policy, focusing on the scientific research paper as a literary genre (eg, the ‘scene’ in Gusfield’s analysis is the scientific journal and the ‘act’ is the research study, which allows partial resolution of ‘trouble’—a scientific problem—described in the paper’s introduction). Both Yanow (2015) and Hajer (2005) applied Burke’s pentad to consider how physical rooms and spaces (‘scene’) influence the deliberative process of policymaking. Hajer, for example, in an analysis of policy-making arguments by the Dutch government around the use of a new piece of land reclaimed from the sea, talks of the dramaturgical dimension of policymaking. Drawing on Burke, he presents ‘*political processes as a sequence of staged performances of conflict and conflict-resolution in a particular setting’* (Hajer 2005, 630).

None of these studies, however, specifically addressed crisis policy. As noted in the ‘Introduction’ section, policymakers and other societal leaders must respond rapidly to a crisis and make sense of what is happening in a timely and ongoing way. They must also present their decisions to the public in a way that is meaningful and legitimate. Indeed, as Boin *et al* put it, ‘*crisis meaning-making makes a crucial difference between obtaining and losing the “permissive consensus” that leaders need to make decisions and formulate policies in times of crisis’* (2016, 79).

Through what we believe to be a novel application of Burke’s pentad, then, we have demonstrated that during a crisis, a mission-critical failure of meaning-making may be surfaced and explored by a close reading and elemental analysis of media narratives. Such an analysis may reveal and *explain* the loss of permissive consensus—in this case, the descent from heroic adventure into political farce.

Notably, the purpose of our study was not to analyse policy but to analyse *media narratives about policy*. We have deliberately not commented on the truth or otherwise of the accounts in our dataset (eg, the pandemic was far from over in July 2020 even though it was depicted by the media as waning). We align with Gabriel (2004) who defines a story as a ‘poetic elaboration of events’—one which convinces not necessarily by its factual accuracy but by its verisimilitude: its ability to resonate with the experience and emotions of the reader. The narratives analysed in this study both reflect and influence the attitudes of both lay people and professionals towards the NHS in general and remote consulting in particular. Broadly speaking, those in period 1 relate to a time, early in the pandemic, when both press and public were confident in the remote by default policy and sought to support it. Those in period 2 relate to a time when many people’s patience with infection control measures was wearing thin and trust in the government was eroding. In this sense, the overarching storylines (worthy battle in period 1 and trouble descending to farce in period 2) were an evocative, if not entirely accurate, reflection of what their readers were experiencing.

The lay media narratives depicted in this study resonated strongly with storylines in the professional literature. The *British Medical Journal* published an editorial the day after the pandemic was declared (12 March 2020) describing video consultations as, potentially, ‘an opportunity in a crisis’—a way of achieving short-term infection control goals and of helping introduce and embed a service model that could have wider benefits (Greenhalgh et al. 2020)Greenhalgh et al. 2020, 998. GPs considered remote consultations essential in the early weeks of the pandemic for infection control reasons (Greenhalgh, Koh, and Car 2020), and such consultations were also depicted as potentially increasing the efficiency of care (Peek, Sujan, and Scott 2020). In June 2020, the *British Journal of General Practice* was still publishing hopeful narratives, asking ‘*how does general practice identify, develop, and embed the positive changes [towards remote care] that are being implemented as a consequence of the crisis?’* (Marshall et al. 2020). But by autumn 2020, remote care had begun to be depicted as potentially damaging to the core values of general practice because it carried risks and threatened the quality of the therapeutic relationship and continuity of care (Gray et al. 2020; Swinglehurst et al. 2020).

The story of remote consulting during and beyond the pandemic is far from over. The scene is changing again: at the time of writing, cases of COVID-19 are rising again and the UK is in its second lockdown. The main actor in period 2, Matt Hancock, depicted by the media as both a villain and even a technophilic fool, will at some stage be replaced by a new Secretary of State. Will his unpopular scheme of modernising the NHS through technology be dropped—or, alternatively, will Hancock’s successor find himself or herself swept up in the prevailing policy wave of techno-solutionism? The drama and its sequel may yet become a trilogy.

## Data Availability

Data are available in a public, open access repository. Dataset is media articles in the public domain as listed in the Appendix.
