# Supplementary material for: ‘From disaster, miracles are wrought’: a narrative analysis of UK media depictions of remote GP consulting in the COVID-19 pandemic using Burke’s pentad
Source: Med Humanit. 2021 Mar 29;47(3):292–301. doi: 10.1136/medhum-2020-012111 (PMC8008912; doi:10.1136/medhum-2020-012111)
Supplement: Supplementary data [file medhum-2020-012111supp001.pdf]

Appendix: Sources used for the narrative analysis

PERIOD 1 (March – June 2020)

Primary dataset

Anonymous, Virtual GP chats are here to stay. *Daily Mail*, May 2, 2020.

Bradley H, Our best defence against coronavirus isn't what you think. *The Independent*, April 15, 2020.

Campbell D, GPs told to switch to digital consultations to combat Covid-19. *The Guardian*, March 6, 2020.

Campbell D, Only one in four GP appointments now conducted in person. *The Guardian*, April 19, 2020.

Chapman B, Coronavirus: Video conferencing apps and remote GP services thrive as world adapts to social distancing. *The Independent*, March 16, 2020.

Epstein A, The medics who can see you now! (But only online). *Daily Mail*, March 31, 2020.

Field M, Doctors using mobile apps to 'see' patients. *The Daily Telegraph*, April 6, 2020.

Francis A, Thousands of patients across Newham get access to remote GP consultations. *The Voice*, April 9, 2020.

Hayward E, Talk to your GP. *Daily Mail*, March 11, 2020.

Lay K, GPs urged to see patients remotely. *The Times*, March 10, 2020.

Lay K, How pandemic is set to future-proof the NHS. *The Times*, April 4, 2020.

Lewis J, Embracing life and taking risks is what being young is about. *The Daily Telegraph*, May 22, 2020.

Libbert L, The doctor will Zoom you now... how the GP surgery went virtual. *The Daily Telegraph*, May 25, 2020.

Lowe Y, Ring GPs, don't visit. *The Sunday Telegraph*, March 15, 2020.

Porter M, Your GP is preparing for the virus. This is how. *The Times*, March 17, 2020.

RobinsonA, As a GP, I see how the challenge of coronavirus could help improve the NHS. *The Guardian*, March 11, 2020.

Sheldrick G, Patients told: Doctor will see you now... but only by video. *The Express*, March 11, 2020.

Sheldrick G, We'll keep you safe. *The Express*, March 14, 2020.

Summerton N, Diary of a GP on the front line. *Daily Mail*, April 14, 2020.

Examples of articles from wider dataset

Borland B, NHS virus revolution. *Daily Mail*, March 18, 2020.

Kelly L, Digital front opens in war on disease as start-ups come to the aid of NHS. *The Sunday Times*, March 29, 2020.

Pietras E, I'm running my surgery like it's the frontline in a war zone. *The Sun*, April 3, 2020.

PERIOD 2 (July 31 – August 13 2020)

Primary dataset

Adams S, My husband was in agony - but GP wouldn't see him due to Covid rules. *Mail on Sunday*, August 2, 2020.

Anonymous, Doctor Zoom. *The Times*, July 31, 2020.

Anonymous, Zoom medicine will be welcomed by many. *The Daily Telegraph*, July 31, 2020.

Borland S, Just 1 in 10 see GP face to face. *Daily Mail*, July 30, 2020.

Burgess K, 'Zoom medicine' is new normal, Hancock insists. *The Times*, July 31, 2020.

Clark R, Seeing the doctor means more than a face on a screen. *The Express*, July 31, 2020.

Cowburn A, All initial GP consultations should now happen on phone or online, Matt Hancock announces. *The Independent*, July 30, 2020.

Davis N, 'Remote first' GP appointments must be handled with care. *The Guardian*, July 30, 2020.

Donnelly L, Video-call appointments with GP may be here to stay. *The Daily Telegraph*, July 30, 2020.

Éclair J, GP video calls aren't all they're cracked up to be. *The Independent*, August 4, 2020.

Hayward E, Why you may never see your family doctor again. *Daily Mail*, July 31, 2020.

Lay K, Only 1 in 10 see their doctor face to face as GPs go online. *The Times*, July 30, 2020.

Phillips F, "Abused NHS is now the one getting sick." *Daily Mirror*, August 1, 2020.

Street-Porter J, 'Zoom medicine' won't work for the elderly - seeing a GP face to face is so important. *The Independent*, August 1, 2020.

Tidman Z, Doctors fear loss of patient relationships as government announces GP appointments to be moved to phone or online. *The Independent*, July 31, 2020.

Walker P, All GP consultations should be remote by default, says Matt Hancock. *The Guardian*, July 30, 2020.

Yorke H, Hancock: video appointments to be the default. *The Daily Telegraph*, July 31, 2020.
